# Psychological resource pathways to life satisfaction in South Africa and India: a cross-national pilot study with implications for employee resilience

**DOI:** 10.3389/fpsyg.2026.1706890

**Published:** 2026-04-14

**Authors:** Anurag Shekhar, Musawenkosi Donia Saurombe

**Affiliations:** Department of Industrial Psychology and People Management, College of Business and Economics, University of Johannesburg, Johannesburg, South Africa

**Keywords:** employee resilience, flourishing, life satisfaction, perceived stress, work engagement, South Africa, India

## Abstract

**Introduction:**

Psychological resources are critical to employee well-being and are widely regarded as enabling resilient functioning in the face of uncertainty. Evidence from non-Western contexts, however, remains limited. This cross-national pilot study examined whether psychological resources, operationalized as a composite of mental well-being (WEMWBS), flourishing (Flourishing Scale), and work engagement (UWES-3), predicted life satisfaction (SWLS) directly and/or indirectly via perceived stress (PSS-4) among employees in South Africa and India. The findings are discussed in relation to their implications for employee resilience theory and resource-building practice.

**Methods:**

An explanatory-sequential mixed-methods design was used. The quantitative phase comprised South African pharmaceutical employees (*n* = 87) and Indian IT employees (*n* = 65), for a total of *N* = 152. After z-standardizing indicators within each country, we formed a Resources composite. We estimated simple mediation (Resources → PSS → SWLS) within each country and a pooled moderation model (standardized SWLS) with Country and interaction terms. The qualitative phase included 2 focus groups and 29 one-on-one interviews (South Africa *n* = 19; India *n* = 10), which were analyzed using reflexive thematic analysis to explain statistical patterns.

**Results:**

Resources showed a strong, positive association with life satisfaction (pooled model: *b* = 0.71, *SE* = 0.11, *p* < 0.001; *R^2^* = 0.358). Resources were linked to lower perceived stress (South Africa: *a* = −0.88, *p* < 0.001; India: *a* = −0.69, *p* = 0.016). However, perceived stress did not uniquely predict life satisfaction once Resources were included (pooled *b* = −0.10, *SE* = 0.10, *p* = 0.331), while bootstrapped indirect effects included zero in both countries. Country did not moderate the relationship between Resources and life satisfaction, nor between perceived stress and life satisfaction (Resources × Country: *p* = 0.165; PSS × Country: *p* = 0.782). Qualitative themes explained the resource-dominant pathway: in South Africa, Ubuntu-based meaning, complex family support, masculine help-seeking norms, and economic/infrastructure strain characterized the resource-dominant pathway; whereas in India, technology-sector pride and aspirational mobility offset AI-related job insecurity, urban infrastructure burdens, and family separation characterized the resource-dominant pathway.

**Discussion:**

The findings support resource-building interventions and motivate future testing of alternative mediators (e.g., meaningful work, positive affect, self-efficacy) through adequately powered longitudinal studies. The cross-country similarity suggests functional equivalence of mechanisms with culturally specific content.

## 1 Introduction

Psychological resources (including mental well-being, positive affect, and work engagement) are central to how employees sustain performance and well-being under occupational demands (Hobfoll et al., 2018; Topino et al., 2022). Such resources are increasingly recognized as foundational antecedents to resilient functioning at work: employees who possess richer psychological resource portfolios are better equipped to adapt to disruption, recover from adversity, and maintain positive functioning over time (Hartmann et al., 2020; Raetze et al., 2021). Theoretically, the Conservation of Resources (COR) theory explains why these resources matter: individuals who accrue valued resources resist stressors more effectively and achieve higher well-being (Hobfoll et al., 2018).

Despite this theoretical foundation, empirical investigations of how psychological resources translate into well-being outcomes—and whether these pathways function equivalently across cultural contexts—remain underexplored, particularly in non-Western settings (Schimmelpfennig et al., 2025). This study addresses this gap by examining whether psychological resources predict life satisfaction directly and/or indirectly through reduced perceived stress among employees in South Africa and India, and whether these pathways are equivalent across the two countries. Although resilience itself is not directly measured, the findings contribute to resilience scholarship by identifying the resource–outcome mechanisms that support resilient employee functioning across culturally distinct emerging economy contexts.

Theoretically, the COR theory explains why resilience matters: individuals who possess or accrue valued resources (e.g., energy, optimism, engagement) are better able to resist stressors and achieve well-being (Hobfoll et al., 2018). In line with this view, studies increasingly show that personal psychological resources are linked to favorable outcomes at work and beyond (Topino et al., 2022; Zhou et al., 2023).

However, the employee resilience literature remains heavily skewed toward Western samples. A recent systematic review of resilience interventions found that Western countries contributed overwhelmingly, highlighting a substantial geographic imbalance in resilience research (Kunzler et al., 2020; Schimmelpfennig et al., 2025). This gap is noteworthy because sociocultural contexts shape both exposure to demands—including work-related demands (workload, time pressure), economic demands (financial strain, job insecurity), social demands (family obligations), and gendered demands (domestic labor expectations)—and available resource pools.

The selection of South Africa and India was motivated by both theoretical and contextual factors. Both countries are major emerging economies within the BRICS grouping, characterized by rapid organizational change, growing knowledge economies, and significant workplace stress (Gallup, 2024; Nach and Ncwadi, 2024)—yet both are substantially underrepresented in the organizational psychology literature (Medvedev et al., 2024; Rad et al., 2018; Schimmelpfennig et al., 2025). This geographic gap is consequential: if resource–well-being pathways are shaped by sociocultural context, findings derived overwhelmingly from Western samples may not generalize to employees’ lived experiences in these settings (Kunzler et al., 2020; Schimmelpfennig et al., 2025).

Beyond their economic comparability, the two countries offer meaningfully distinct cultural frameworks for understanding psychological resources. South Africa is characterized by Ubuntu philosophy—a relational, communal worldview emphasizing collective identity, interdependence, and shared humanity—which is likely to shape how social and psychological resources are experienced and mobilized (Pillay, 2023; Theron and Theron, 2010). India, and particularly its technology sector, is shaped by aspirational mobility narratives, a family-oriented collectivism of a different kind, and rapid digital-economic transformation that creates both resource-generating opportunities and novel stressors (Fenn et al., 2021; Premchandran and Priyadarshi, 2018). Examining resource–outcome pathways in both contexts simultaneously allows us to test whether COR theory mechanisms demonstrate functional equivalence across culturally distinct yet economically comparable settings—a question with significant implications for multinational organizations and global well-being policy.

Furthermore, African organizational contexts are particularly neglected in the broader literature. A recent audit of organizational behavior research found that African samples represent a very small percentage of published studies, a disparity that constrains both theoretical development and practical application (Hendriks et al., 2018; Kunzler et al., 2020; Schimmelpfennig et al., 2025). Similarly, the field of well-being research in India remains limited (Hendriks et al., 2018; Pangtey et al., 2020; Schimmelpfennig et al., 2025; Traymbak et al., 2022); studies examining cross-national pathways linking psychological resources to life satisfaction through COR theory are similarly limited.

To address this gap, the present cross-national pilot research investigated a simple, theory-driven pathway in two understudied settings: South Africa and India. These countries represent culturally distinct yet economically comparable emerging-market contexts in which workplace stress and employee well-being are increasingly relevant to organizational sustainability and economic development. We conceptualize psychological resources as a composite of three established indicators: general mental well-being (Warwick–Edinburgh Mental Well-Being Scale; WEMWBS), flourishing (Flourishing Scale), and work engagement (UWES-3). Each is widely used and psychometrically supported, including in multi-country research (Blodgett et al., 2022; Diener et al., 2010; Maheswaran et al., 2012; Schaufeli and Bakker, 2003). These three constructs represent complementary facets of psychological functioning that collectively capture an individual’s resource portfolio: eudaimonic well-being (flourishing), hedonic and eudaimonic well-being (WEMWBS), and work-specific vigour and dedication (UWES). On theoretical grounds, those resources should be associated with better global evaluations of life (life satisfaction) and potentially operate *via reduced perceived stress,* consistent with COR dynamics in which resources buffer stress exposure and appraisals (Hobfoll et al., 2018; Topino et al., 2022).

Life satisfaction is an established indicator of subjective well-being and a meaningful outcome for employees and organizations alike, reflecting broad cognitive judgments of one’s life as a whole (Diener et al., 1985). Perceived stress captures the extent to which situations are appraised as overwhelming or uncontrollable (Cohen et al., 1983) and is typically inversely related to well-being. Recent evidence across employee and community samples continues to link resilience-related resources to higher life satisfaction, higher work engagement, and lower stress (Cazan and Camelia, 2015; Zheng et al., 2020; Zhou et al., 2023). Theoretically, the stress-buffering function of psychological resources suggests that their influence on life satisfaction may operate primarily through enhanced coping and reduced threat appraisals, rather than solely through direct positive affect pathways (Cohen et al., 1983). However, whether stress mediates the relationship between resources and life satisfaction—and whether this pathway is comparable across countries—remains insufficiently tested (Zheng et al., 2020).

### 1.1 Research gap and contribution

Existing literature leaves at least three important gaps that this study addresses. First, although COR theory has been extensively applied to Western samples, empirical tests of resource–stress–life satisfaction pathways that explicitly compare South African and Indian employees are absent from the literature. Although Medvedev et al. (2024) explicitly compare India and South Africa, their study focuses on monetary *versus* psychological incentives rather than the specific “resource–stress–life satisfaction” COR pathway. Most cross-cultural resilience and well-being research either treats “non-Western” as a monolithic category or focuses on a single country, precluding direct cross-national comparison (Schimmelpfennig et al., 2025).

Second, prior studies have largely examined constructs such as mental well-being, flourishing, and work engagement as separate predictors of well-being outcomes (Rothmann, 2008), rather than as a theoretically coherent composite resource portfolio. By aggregating these three established indicators into a composite index informed by COR theory, this study tests whether a holistic resource portfolio—rather than any single resource—predicts life satisfaction and operates via stress-reduction pathways.

Third, and critically, the role of perceived stress as a mediator between psychological resources and life satisfaction has been assumed rather than empirically tested in many non-Western contexts (Bandeira et al., 2023; Zheng et al., 2020). Whether resources operate primarily by reducing stress (a sequential mediation pathway) or directly (through meaning and purpose pathways) has important implications for both COR theory and the design of workplace interventions in emerging economy settings.

This study addresses these gaps through an explanatory-sequential mixed-methods pilot design combining quantitative pathway testing with qualitative mechanism exploration. The mixed-methods integration allows us not only to test whether the mediation and moderation effects hypothesized by COR theory are supported, but also to explain the mechanisms through which resources influence life satisfaction when the hypothesized stress pathway is not supported—contributing to theoretical refinement rather than merely confirming or disconfirming existing models.

## 2 A brief literature review

### 2.1 Conservation of resources theory and employee resilience

The COR theory provides the foundational framework for understanding mechanisms of employee resilience. Over the past three decades, COR theory has become one of the most widely cited theories in organizational psychology, serving as the theoretical basis for the Job Demands-Resources model (Hobfoll et al., 2018). The theory posits that individuals strive to obtain, retain, and protect valued resources, with resource depletion leading to stress and negative outcomes, whereas resource accumulation promotes well-being and adaptive functioning.

Recent applications of COR theory demonstrate its continued relevance for understanding employee well-being. Brady et al. (2025) found that supervisor resilience promotes employee well-being through resource crossover mechanisms, suggesting that resilience functions as a transferable resource within organizational contexts. Similarly, Wang and Wang (2024) indicate that employee resilience can influence organizational resilience in construction projects, with the relationship mediated by team task characteristics, supporting COR theory’s prediction that individual resources contribute to collective outcomes.

### 2.2 Cross-cultural research gaps

Despite the widespread application of the COR theory, noteworthy geographical imbalances persist in resilience research. Kunzler et al. (2020) conducted a comprehensive systematic review comparing resilience interventions across Western and Eastern countries, finding that Western countries contributed more substantially to the development of resilience interventions than Eastern countries. This geographic concentration constrains understanding of how resilience mechanisms may vary across cultural and economic contexts, particularly in emerging economies, where workplace stressors and resource availability may differ substantially from those in developed Western contexts.

The tourism industry research by Prayag et al. (2024) in Sri Lanka illustrates the importance of contextual factors in resilience processes. Their study revealed that resilient leadership behaviors enhanced both employee and organizational resilience during the COVID-19 crisis, although the unique cultural and economic contexts of the Sri Lankan tourism sector shaped the specific mechanisms. This highlights the need for cross-cultural research to explore how COR theory mechanisms operate, vary, or adapt across diverse cultural and economic settings, rather than assuming universal applicability (Schimmelpfennig et al., 2025). Such research examines whether theoretical processes demonstrate functional equivalence (similar mechanisms with different content) or require context-specific modification.

### 2.3 Mediation mechanisms in employee well-being

Traditional stress-and-coping models position stress reduction as the primary mechanism by which personal resources enhance well-being outcomes. However, emerging evidence suggests that alternative pathways may be equally or more important (Arnold et al., 2007; Csordás et al., 2022). Recent research indicates that meaningful work and positive affect may serve as key mediators between personal resources and life satisfaction, particularly in professional populations with moderate rather than extreme baseline stress levels (Arnold et al., 2007; Raetze et al., 2021).

The application of COR theory to remote work contexts further illustrates the complexity of resource–outcome pathways. Flexible work arrangements, autonomous work, and time-saving resources support work-life balance (which might be through mechanisms involving self-efficacy and supervisor trust) rather than solely through stress reduction (Kelliher and Anderson, 2010; Wood, 2008). This suggests that resources may operate through multiple simultaneous pathways rather than through sequential mediation chains.

### 2.4 Theoretical integration and research imperatives

Current theoretical developments in organizational psychology emphasize the need for models that accommodate both cultural universality and contextual specificity in resilience and well-being processes (Schimmelpfennig et al., 2025). Although COR theory provides a robust foundation for understanding resource dynamics across contexts (Hobfoll et al., 2018), its application in culturally diverse settings requires empirical validation rather than relying solely on theoretical assumptions (Hobfoll et al., 2018; Kunzler et al., 2020; Schimmelpfennig et al., 2025). The limited representation of African and South Asian organizational contexts in the literature represents a significant knowledge gap that constrains both theoretical development and practical application (Prayag et al., 2024; Schimmelpfennig et al., 2025).

This study addresses these gaps by examining COR theory’s predictions in South African and Indian organizational contexts, testing whether resource–outcome pathways demonstrate functional equivalence across culturally distinct emerging economy settings. By employing mixed-methods approaches that combine quantitative pathway testing with qualitative mechanism exploration, this research contributes to the development of culturally informed and theoretically coherent models of employee resilience.

### 2.5 COR theory and resource–well-being pathways in non-Western contexts

Although COR theory was developed in Western contexts, emerging evidence supports its cross-cultural applicability, though the valuation of specific resources often depends on cultural beliefs and values (Hobfoll et al., 2018). In Asian contexts, research among Chinese healthcare workers shows that personal resources, such as resilience, significantly predict work engagement and protect against burnout (Guo et al., 2017; Zhou et al., 2023). Furthermore, studies in China suggest that specific cultural values emphasizing relationships and social identification serve as critical antecedents to individual resilience and organizational commitment (Wei and Taormina, 2014; Zhou et al., 2023).

In South Asian contexts, evidence from Sri Lanka indicates that resilient leadership behaviors—such as vision sharing and change management—serve as vital organizational resources that foster both employee and organizational resilience during crises (Prayag et al., 2024). Within India, personal resources, including self-efficacy and positive coping strategies, are recognized as essential enablers of well-being for professionals in high-stress sectors, such as mental health and social work (Bhagwagar, 2022; Stanley and Mettilda Bhuvaneswari, 2016). Additionally, research among Indian medical students suggests that, although the relationships between resilience and well-being are complex, resilience enables greater autonomy and purpose (Sonika et al., 2019).

Evidence from African organizational contexts reinforces the need for more representative research. In South Africa, psychological resources such as emotional intelligence have been found to predict career adaptability among Black call center agents, a population facing high levels of uncertainty and emotional labor (Coetzee and Harry, 2014). Similarly, Rothmann (2008) established that dimensions of work-related well-being, including work engagement and burnout, are independent but related factors influencing the performance of South African police officers. The cultural philosophy of “Ubuntu” is also recognized as a uniquely African resource that facilitates collective resilience through shared psychosocial support (Pillay, 2023; Theron and Theron, 2010).

Despite these insights, a substantial gap remains because approximately 86% of articles in top management journals utilize samples from “WEIRD” (Western, Educated, Industrialized, Rich, and Democratic) contexts (Rad et al., 2018; Schimmelpfennig et al., 2025). This sampling bias limits the generalizability of resource–well-being pathways to low- and middle-income regions such as sub-Saharan Africa, where healthcare workers and other professionals often report lower resilience prevalence than global standards (Mcizana et al., 2024; Schimmelpfennig et al., 2025). This study builds on this foundation by providing a direct comparison between these understudied settings.

### 2.6 Research hypotheses

Building on the theoretical and empirical foundations outlined above, we formulate three hypotheses:

*Hypothesis 1*: COR theory proposes that individuals with richer psychological resources are better positioned to evaluate their lives positively, as resources enable effective functioning across life domains (Hobfoll et al., 2018). Cross-cultural evidence consistently supports positive associations between psychological resources and life satisfaction in both Western and emerging economy contexts (Rothmann, 2008; Topino et al., 2022; Zhou et al., 2023). We therefore predict:

*Hypothesis 1*: Psychological resources (operationalised as a composite of mental well-being, flourishing, and work engagement) will be positively associated with life satisfaction in both South African and Indian employees.

*Hypothesis 2*: A core COR prediction is that resources buffer against stress appraisals, reducing perceptions of uncontrollability (Hobfoll et al., 2018). Perceived stress, in turn, consistently predicts lower subjective well-being (Warttig et al., 2013). This suggests a sequential pathway in which psychological resources enhance life satisfaction partly by reducing perceived stress—a stress-buffering mechanism aligned with traditional coping models. We therefore predict:

*Hypothesis 2*: The relationship between psychological resources and life satisfaction will be partially mediated by perceived stress, such that higher psychological resources will be associated with lower perceived stress, which in turn will be associated with higher life satisfaction in both South African and Indian employees.

*Hypothesis 3*: Despite the theoretical universality of COR mechanisms, cultural context shapes which resources are valued and how they convert into well-being outcomes (Prayag et al., 2024; Schimmelpfennig et al., 2025). Given substantial differences between Ubuntu-informed collectivism in South Africa and aspirational individualism–collectivism in India, pathway strengths may differ. However, given limited prior cross-national evidence from these specific contexts, this remains an exploratory hypothesis:

*Hypothesis 3*: The strength of the direct and indirect pathways from psychological resources to life satisfaction will differ significantly between South African and Indian participants, reflecting cultural and contextual variations in resilience mechanisms.

In line with the pilot scope, we also examined whether the strengths of these paths differ across countries, without strong *a priori* expectations given the limited cross-national evidence from emerging-market contexts.

## 3 Methodology

### 3.1 Study overview

To address the identified research gaps, we conducted an explanatory-sequential mixed-methods study (Creswell and Clark, 2017) examining resilience pathways in South African and Indian employees. This design allows quantitative pathway testing followed by qualitative explanation of observed patterns—an approach particularly valuable when exploring mechanisms in understudied cultural contexts where existing theory may require refinement (Fetters et al., 2013).

This cross-sectional pilot study forms part of a larger longitudinal employee well-being intervention conducted across both countries. The present investigation serves multiple purposes: establishing baseline resilience pathways, testing measurement feasibility across cultural contexts, and identifying country-specific stressors to inform subsequent intervention design. We recruited participants from a pharmaceutical company in South Africa and an information technology company in India, representing distinct yet comparable organizational contexts in the emerging knowledge economy.

### 3.2 Participants

#### 3.2.1 Quantitative phase

The final sample comprised 152 knowledge workers from two organizations: 87 participants (57.2%) from a South African company and 65 participants (42.8%) from an Indian company. Table 1 presents the demographic characteristics by country, revealing important cross-national differences.

**Table 1:** Participant demographic characteristics by country.

Characteristic	South Africa (*n* = 87)	India (*n* = 65)
Sex		
Female	63 (72.4%)	28 (43.1%)
Male	24 (27.6%)	37 (56.9%)
Age		
Range	27–61 years	22–38 years
*M* (SD)	44.13 (7.88)	26.80 (3.20)
Education		
Postgraduate	21 (24.1%)	49 (75.4%)
Bachelor’s	24 (27.6%)	16 (24.6%)
Secondary school or below	42 (48.3%)	0 (0.0%)
Income		
Above average	26 (29.9%)	10 (15.4%)
Average	54 (62.1%)	46 (70.8%)
Below average	5 (5.7%)	9 (13.8%)
Affluent	2 (2.3%)	0 (0.0%)
Race/Ethnicity		
White	47 (54.0%)	0 (0.0%)
Black	18 (20.7%)	0 (0.0%)
Indian/Asian	16 (18.4%)	65 (100.0%)
Colored	6 (6.9%)	0 (0.0%)

The samples differed substantially in age composition. South African participants ranged in age from 27 to 61 years (*M* = 44.13, SD = 7.88), while Indian participants ranged from 22 to 38 years (*M* = 26.80, *SD* = 3.20). Sex distribution also varied: the South African sample was predominantly women (72.4%), whereas the Indian sample was predominantly men (56.9%).

Educational attainment differed markedly between countries. In the Indian sample, 75.4% held postgraduate qualifications compared to 24.1% in South Africa. By contrast, 48.3% of South African participants had completed secondary education or below, while all Indian participants held at least a bachelor’s degree. This pattern reflects sectoral differences, with the Indian sample drawn from technology/IT sectors requiring advanced technical qualifications.

Income distributions were relatively similar across countries, with most participants reporting average income levels (South Africa: 62.1%; India: 70.8%). However, the South African sample included slightly more above-average earners (29.9% *vs*. 15.4%) and two affluent participants.

The South African sample demonstrated racial diversity reflecting the country’s demographics: 54.0% White, 20.7% Black, 18.4% Indian/Asian, and 6.9% Colored participants—all participants in the Indian sample identified as Indian/Asian, consistent with the Mumbai metropolitan location.

#### 3.2.2 Qualitative phase

We conducted 2 focus group discussions (FGDs) (19 participants in the South African FGD and 14 in the Indian FGD) and 29 individual interviews (19 in South Africa and 10 in India). Participants were recruited from the same organizations but were independent of the quantitative sample. We used maximum-variation purposive sampling to ensure diversity across sex, tenure, and job function. We report the qualitative component in accordance with the Consolidated Criteria for Reporting Qualitative (COREQ) 32-item checklist; the completed checklist is provided as Supplementary Table S1.

### 3.3 Research procedure

#### 3.3.1 Quantitative data collection

Data were collected through an online English-language survey. Each organization’s HR department distributed the survey link internally. Average completion time was 20–30 min. Participants provided informed consent electronically before beginning the survey.

#### 3.3.2 Qualitative data collection

Focus groups and interviews lasted 60–75 min and were conducted in English. Focus group participants requested that the sessions not be recorded to encourage candid responses. Detailed notes were thus taken and subsequently anonymized. The semi-structured interview guide used in both FGDs and one-on-one interviews explored five main areas: salient job demands over the past 12–18 months; stress experiences and coping strategies; personal and organizational resources (such as engagement and social support); perceived effects on life satisfaction; and reflections on recent organizational changes.

The research aim, literature on well-being, and workplace well-being interventions informed the guide. It comprised open-ended questions designed to encourage participants to share their perspectives while allowing the interviewer to probe deeper into specific themes. The interview guide ensured that all key topics were covered while also enabling participants to discuss aspects that were personally meaningful to them.

### 3.4 Measures

#### 3.4.1 Psychological resources composite

We assessed three constructs and combined them after standardization to represent overall psychological resources:

The Warwick–Edinburgh Mental Well-Being Scale (WEMWBS) comprises 14 items, each scored on a 5-point Likert scale. The total score on the scale ranges from 14 to 70, with higher scores indicating higher levels of mental health (Taggart et al., 2013). It has good validity and high levels of internal consistency (Cronbach’s alpha ranging from 0.89 to 0.91) and test–retest reliability of 0.83 (Tamminen et al., 2020). It measures positive aspects of mental health (the measure of mental well-being), covering both hedonic and eudaimonic aspects of mental health, including positive affect (e.g., optimism, cheerfulness, and relaxation), the robustness of relationships, and positive functioning (e.g., energy, clear thinking, autonomy, personal acceptance, etc.) (Tennant et al., 2007). It shows a strong correlation with other well-being instruments and does not exhibit a ceiling effect in the population sample (Tennant et al., 2007). This tool has performed well in the multicultural societies of South Africa and India (Grover and Dua, 2021; Shekhar et al., 2025a; Smith, 2018).

The Flourishing Scale is a brief 8-item measure of an individual’s self-perceived success in important areas, including relationships, optimism, self-esteem, and purpose (Diener et al., 2018b). It results in a single psychological well-being score. The concept of flourishing represents more eudaimonic well-being (Choudhry et al., 2018; Tan et al., 2021). This tool demonstrates strong psychometric properties with an internal consistency of 0.87 and temporal reliability of 0.71 (Checa et al., 2017). The scale was also tested in South Africa and India (Mostert et al., 2023; Singh et al., 2018). The scale’s internal consistency is high, with Cronbach’s alpha typically reported at 0.87–0.91, indicating excellent reliability. Test–retest reliability over short intervals has been similarly strong (Diener et al., 2010).

The Utrecht Work Engagement Scale (UWES) is one of the most widely used self-report questionnaires for measuring work engagement. It assesses three constructs: vigour, dedication, and absorption, collectively defining engagement as a positive, fulfilling, and work-related state of mind (Schaufeli and Bakker, 2004). Vigour refers to high levels of energy and resilience while working, dedication indicates a sense of significance and enthusiasm, and absorption reflects being fully engrossed in work and able to concentrate. The UWES is available in 17-, 9-, and 3-item versions and has been validated in multiple languages, demonstrating its versatility across diverse cultural contexts.

UWES is one of the most popular measures of engagement, alongside the Gallup Q12. It has been translated into multiple languages and used across many professions (Seppala et al., 2009). Studies have also confirmed the reliability and validity of the ultra-short scale UWES-3 (Choi et al., 2020; Lazauskaitė-Zabielskė et al., 2020). The Cronbach’s alpha for work engagement was 0.776, indicating acceptable internal consistency, while the convergent validity was significant (correlation coefficient: 0.42) for the 3-item scale (Seppala et al., 2009). The UWES scale also shows correlations with job resources and job satisfaction, while the correlation with burnout and job demands is negligible. Overall, the ultra-short version of UWES-3 shows excellent psychometric properties (Merino et al., 2021; Su et al., 2023). The UWES has been tested in South Africa and India (Agarwal et al., 2012; Storm and Rothmann, 2003).

Each scale was standardized within each country and then averaged to create the psychological resources composite. Higher scores indicate greater psychological resources.

#### 3.4.2 Life satisfaction

Subjective well-being has two parts: affect and the cognitive evaluation of one’s life. Affect can change, but satisfaction with life (which represents cognitive evaluation) is more stable (Diener et al., 1985, 2018a; Townshend, 2023). This scale (the Satisfaction with Life) was developed by Ed Diener and his colleagues in 1985 to measure life satisfaction. It briefly assesses an individual’s general sense of life satisfaction.

It has been used in many studies related to well-being (Townshend, 2023). This scale is associated with the mental components of quality of life, anxiety, optimism, and sleep quality (Hinz et al., 2018). The Satisfaction with Life Scale (SWLS) is among the best-suited scales for non-clinical populations (Townshend, 2023). The SWLS generally demonstrates high internal consistency, with a Cronbach’s alpha ranging from 0.79 to 0.89, indicating good reliability. Test–retest reliability has also been reported, with correlations ranging from 0.82 to 0.87 over periods of up to 2 months (Diener et al., 1985). This scale has been tested in South Africa and India (Schutte et al., 2021; Traymbak et al., 2022).

#### 3.4.3 Perceived stress

The Perceived Stress Scale (PSS) is one of the most widely used instruments to assess psychological stress (Figalová and Charvát, 2021; Lee, 2012; Ruisoto et al., 2020; Warttig et al., 2013). Multiple studies have confirmed its reliability and validity in different languages (Warttig et al., 2013). The internal consistency of the items was strong (*r*-values ranging from 0.84 to 0.86), and it has shown a test–retest reliability of 0.85 (Roberti et al., 2006). The original instrument has 14 statements that measure the extent to which respondents feel their lives are unpredictable, uncontrollable, and overloaded (Warttig et al., 2013). The two shorter PSS versions have 10 and 4 items. PSS-4 was developed for rapid data collection, with only four items, making it less time-consuming (Du et al., 2023). All three versions of the PSS tool show a satisfactory fit to the item structure, with PSS-4 recommended for quick and large-sample use (Ruisoto et al., 2020). All versions of the PSS have demonstrated good internal consistency and stability over time (Figalová and Charvát, 2021). Studies have found similarities in the psychometric properties of PSS-10 and PSS-4 and have shown that the two scales are highly correlated (Figalová and Charvát, 2021). PSS-4 is a reliable and valid tool for assessing stress in university-educated participants (Sanabria-Mazo et al., 2023). The scores on the above four questions range from 0 to 16, with higher scores indicating higher stress levels. A score of 6 or higher indicates that the participant is experiencing a high level of perceived stress (Warttig et al., 2013). PSS has been widely used in South Africa and India (Blignaut et al., 2022; Cristóbal-Narváez et al., 2020; Fenn et al., 2021; Mozumder, 2017; Pangtey et al., 2020).

#### 3.4.4 Demographics

In addition to assessing participants’ well-being, this study collected demographic data, including age, sex, race, and education level, to explore potential influences on various aspects of well-being. These variables were collected via a self-reported questionnaire to ensure comprehensive analysis and control of potential confounding factors. Alongside these demographic factors, measures of work-related stress and engagement were collected to provide a more holistic understanding of participants’ well-being and its potential relationship with occupational factors. This allowed for a deeper examination of how personal and work-related variables shape resilience and overall well-being.

### 3.5 Data analysis

#### 3.5.1 Quantitative analysis

Analyses were conducted using SPSS version 28. We calculated descriptive statistics, reliability coefficients, and Pearson correlations for all variables. The primary analysis tested a simple mediation model with three components:

- Total effect of psychological resources on life satisfaction
- Effect of psychological resources on perceived stress (a-path)
- Effects of both psychological resources and perceived stress on life satisfaction (b-path and direct effect)

We calculated indirect effects using bootstrap resampling with 5,000 resamples and 95% confidence intervals. We then tested a moderation model to examine whether relationships differed by country.

Model assumptions were verified through residual diagnostics, including tests for linearity, homoscedasticity, and normality. Missing data were minimal (<2%) and handled by casewise deletion. The final analytic sample was 152 participants. All significance tests used a two-tailed alpha of 0.05.

#### 3.5.2 Common method bias

Given the single-source self-report design, common method bias (CMB) was considered a potential threat to validity (Podsakoff et al., 2003). Procedural remedies included independent survey administration through the university’s statistical department, with data flowing directly to the research team rather than organizational management. Participants were assured of strict confidentiality, reducing evaluation apprehension. Additionally, different response formats across scales (frequency *vs*. agreement) reduced uniform acquiescence bias.

Harman’s single-factor test was conducted *post hoc* by entering all scale items into an exploratory factor analysis. The unrotated solution yielded multiple factors, with the first factor accounting for 34.7% of total variance—substantially below the 50% threshold indicative of severe CMB (Podsakoff et al., 2003). Although these safeguards reduce CMB concerns, single-source cross-sectional limitations remain and are acknowledged in Section 5.6.

The analysis was conducted using Python 3.12 with scikit-learn 1.8.0 for principal component analysis. The analysis script is available from the corresponding author upon request to ensure full replicability.

#### 3.5.3 Qualitative analysis

We used reflexive thematic analysis (Braun and Clarke, 2019) to identify patterns within and across countries. The primary researcher independently coded an initial subset of data. Both researchers then met to develop consensus coding and refine the codebook using both inductive and deductive approaches aligned with the quantitative model. The full dataset was then coded systematically. We maintained detailed audit trails through memos and decision logs.

#### 3.5.4 Trustworthiness

Qualitative rigour was ensured through several strategies. Credibility was enhanced through analyst triangulation and informal resonance checks, whereby emerging theme summaries were reviewed against participant accounts and field notes during iterative coding. Dependability was maintained through detailed audit trails documenting coding decisions and analytical procedures. Transferability was supported through a rich description of participant characteristics and organizational contexts.

#### 3.5.5 Reflexivity and positionality

The focus group discussion and all interviews were conducted by the first author. At the time of the research, the interviewer was employed by one of the organizations and obtained prior approval from both the company and the university ethics committee. Aware of his positionality as a male, Asian, and manager, he maintained a reflexive journal, clarified his non-evaluative role, and emphasized confidentiality and voluntariness to mitigate power dynamics. Furthermore, his formal training in coaching was consciously employed to foster a psychologically safe environment, using active listening and open-ended questioning to encourage participants to engage and contribute deeply. To mitigate power dynamics and social desirability bias, sessions were held in neutral venues; no direct reports were recruited; participation was voluntary with the right to withdraw; and confidentiality was emphasized through independent survey administration. Immediate post-interview field notes and iterative memoing documented the potential influence of the researcher’s sex, ethnicity, and managerial role on questioning and interpretation. The insider position in one of the companies also facilitated ongoing informal check-ins and discussions after the formal interviews, yielding additional contextual insights that enriched the analysis. We treat the insider position as a source of both access (trust, contextual sensitivity) and bias; interpretations were therefore checked against disconfirming cases and peer debriefed with academic colleagues. Residual influence of positionality remains possible and is acknowledged as a limitation.

#### 3.5.6 Mixed-methods integration and triangulation

Integration employed methodological triangulation (Denzin, 2009) by combining quantitative mediation analysis with qualitative thematic exploration, allowing us to both test and explain resource–outcome pathways. Data triangulation was achieved through cross-national sampling, enabling pattern comparison across South African and Indian contexts. Analytical rigour was enhanced through collaborative coding, with both authors discussing coding decisions and refining the codebook iteratively to reduce single-researcher bias.

The explanatory-sequential design enabled the qualitative phase to target explanations for quantitative patterns observed (Creswell and Clark, 2017). We systematically linked statistical pathways with qualitative themes through a joint display (Table 2), developing meta-inferences that emphasize resource-dominant pathways and propose alternative mechanisms for future testing.

**Table 2:** Quantitative–qualitative triangulation matrix.

Quantitative pattern	Qualitative explanation	Theoretical implication
Strong resources → life satisfaction pathway across countries	SA: Ubuntu-based meaning; IND: Technology-sector pride and aspirational mobility	Resources operate directly through meaning and purpose rather than stress reduction
Significant resources → stress association but non-significant stress → satisfaction pathway	Ambient stress normalization; constrained disclosure (masculine norms); stress as “background noise”	Stress functions as a parallel rather than a mediating process in this population
Sex differences in well-being (SA)	Gendered domestic labor; single parenthood pressures; extended family obligations	Structural factors moderate individual resource–outcome relationships
Cross-cultural consistency in pathway strength	Ubuntu (SA) and aspirational pride (IND) serve equivalent meaning-making functions	Cultural content differs but psychological mechanisms show functional equivalence

### 3.6 Ethical considerations

The University of Johannesburg’s Research Ethics Committee approved this study (Ethics Clearance Code: IPPM-2022-618(D)). All participants provided written informed consent and were informed of their right to withdraw without consequence. Confidentiality was maintained through anonymized responses and secure data storage. Although HR departments facilitated recruitment, they had no access to individual participant data. The study complied with South Africa’s Protection of Personal Information Act and equivalent data protection standards in India.

## 4 Findings

### 4.1 Preliminary analyses and sample characteristics

The final analytic sample comprised 152 participants (South Africa, *n* = 87; India, *n* = 65) after listwise deletion of missing data (<2%). Internal consistency for the psychological resources composite (calculated from three standardized component scales: WEMWBS, Flourishing Scale, UWES-3) was acceptable in both countries (South Africa, *α* = 0.858; India, α = 0.837) (see Table 3). Variance inflation factors remained below 2.5 across all models, indicating no multicollinearity concerns.

**Table 3:** Sample characteristics and reliability by country.

Country	*n*	α Resources (WEMWBS+FS+UWES)
South Africa	87	0.858
India	65	0.837
Total	152	

Composite reliability and average variance extracted for each scale are reported in Supplementary Table S3. Four of the five scales met the recommended composite reliability (CR) threshold of 0.70, and three met the AVE threshold of 0.50; PSS-4 fell below both thresholds, reflecting the known measurement limitations of the brief 4-item version.

Standardized confirmatory factor analysis (CFA) factor loadings for all scales are presented in Supplementary Table S4. Most items demonstrated acceptable loadings (*λ* > 0.40), except for PSS-4 Item 3 (λ = 0.22).

### 4.2 Descriptive statistics

Table 4 presents descriptive statistics and between-country comparisons for all study variables. Welch’s *t*-tests revealed no statistically significant differences between South African and Indian participants on any measure, *though Indian participants showed marginally higher mental well-being scores (WEMWBS: p = 0.083, d = 0.29).* Effect sizes were small to negligible (Cohen’s *d* range: −0.02–0.29), with confidence intervals that encompassed zero, indicating substantial similarity across the two samples despite their different organizational sectors.

**Table 4:** Descriptive statistics and group comparisons.

Measure	SA *M (SD)*	India *M (SD)*	Welch *t*	*p*	Cohen’s *d*	*d* 95% CI
WEMWBS	48.86 (7.98)	51.12 (7.82)	1.748	0.083	0.286	[−0.04, 0.61]
Flourish	45.60 (5.82)	46.08 (5.96)	0.495	0.621	0.081	[−0.24, 0.40]
UWES	4.43 (1.13)	4.68 (0.89)	1.567	0.119	0.248	[−0.08, 0.57]
PSS	6.00 (1.94)	6.14 (2.03)	0.424	0.672	0.070	[−0.25, 0.39]
SWLS	23.11 (6.38)	22.97 (5.39)	−0.152	0.879	−0.024	[−0.35, 0.29]

Positive *t* and *d* values indicate India > South Africa (SA); negative values indicate South Africa > India. All group differences are non-significant (*p* > 0.05). WEMWBS, Warwick–Edinburgh Mental Well-Being Scale; Flourishing, Flourishing Scale; UWES-3, Utrecht Work Engagement Scale (3-item); PSS-4, Perceived Stress Scale (4-item); SWLS, Satisfaction with Life Scale.

### 4.3 Intercorrelations among study variables

Zero-order correlations among study variables are presented in Table 5. The three psychological resource components demonstrated strong intercorrelations (*r*s = 0.58–0.76), supporting their aggregation. Composite resources correlated positively with life satisfaction (*r* = 0.52, *p* < 0.001) and negatively with perceived stress (*r* = −0.38, *p* < 0.001). These patterns were consistent across countries: resources–satisfaction correlations were 0.64 (South Africa) and 0.50 (India); resources–stress correlations were −0.40 (South Africa) and −0.30 (India).

**Table 5:** Intercorrelations among study variables and discriminant validity.

Particular	$\sqrt{\mathrm{AVE}}$	1	2	3	4
WEMWBS	0.635				
SWLS	0.740	0.515**			
Flourish	0.660	0.762**	0.587**		
UWES	0.784	0.616**	0.430**	0.579**	
PSS	0.482	−0.376**	−0.278**	−0.284**	−0.261**

*N* = 152. √AVE = square root of average variance extracted (for discriminant validity assessment). **p < 0.01. Diagonal values (bold) = square root of average variance extracted (√AVE). The Fornell–Larcker criterion is met when √AVE exceeds all off-diagonal correlations in the same row/column.

### 4.4 Primary mediation analysis

Hayes’ PROCESS Model 4 with 5,000 bootstrap resamples tested the hypothesized mediation pathway from psychological resources to life satisfaction via perceived stress. The results, presented in Table 6, revealed a robust total effect of psychological resources on life satisfaction (*c* = 3.99, SE = 0.45, *p* < 0.001, 95% CI [3.10, 4.88]). This substantial relationship indicates that employees with higher psychological resources consistently report greater life satisfaction across both cultural contexts.

**Table 6:** Mediation analysis—full regression output.

A. Outcome variable: perceived stress (PSS)						
Predictor	B	SE	t	*p*	LLCI	ULCI
Constant	6.0592	0.1502	40.3492	<0.001	5.7625	6.3559
Resources (X)	−0.8028	0.1724	−4.6560	<0.001	−1.1435	−0.4621

Model summary						
R	R-squared	MSE	F	df1	df2	*p*
0.3553	0.1263	3.4277	21.6784	1	150	<0.001

B. Outcome variable: life satisfaction (SWLS)						
Predictor	B	SE	t	*p*	LLCI	ULCI
Constant	24.5183	1.3531	18.1202	<0.001	21.8446	27.1921
Resources (X)	3.7935	0.4828	7.8580	<0.001	2.8395	4.7474
PSS (M)	−0.2419	0.2137	−1.1320	0.2594	−0.6641	0.1803

Model summary						
R	R-squared	MSE	F	df1	df2	*p*
0.5895	0.3476	23.4772	39.6871	2	149	<0.001

C. Total, direct, and indirect effects						
Effect type	B	SE	t	*p*	LLCI	ULCI
Total effect (c)	3.9877	0.4517	8.8287	<0.001	3.0952	4.8801
Direct effect (c’)	3.7935	0.4828	7.8580	<0.001	2.8395	4.7474

Indirect effect (bootstrap 5,000 resamples)					
Effect path	Effect	Bootstrap SE	Bootstrap LLCI	Bootstrap ULCI	Interpretation
via PSS	0.1942	0.1990	−0.1686	0.6158	Not significant (CI includes zero)

Analysis based on Hayes’ PROCESS Model 4 with 5,000 bootstrap resamples. Bootstrap confidence intervals use the percentile method. PSS, Perceived Stress Scale (4-item); SWLS, Satisfaction with Life Scale; Resources, z-standardized composite of WEMWBS + Flourishing Scale + UWES-3.

Examining the individual pathways, the a-path from resources to stress was significant and negative (*a* = −0.80, SE = 0.17, *p* < 0.001, 95% CI [−1.14, −0.46]), confirming that higher psychological resources were associated with lower perceived stress levels. This finding aligns with COR theory predictions that resource-rich individuals experience reduced threat appraisals and enhanced coping capacity.

However, the critical b-path from stress to life satisfaction, while controlling for resources, failed to reach statistical significance (*b* = −0.24, SE = 0.21, *p* = 0.259, 95% CI [−0.66, 0.18]). This unexpected finding suggests that, once psychological resources are accounted for, perceived stress does not uniquely contribute to life satisfaction. The direct effect of psychological resources on life satisfaction remained substantial when controlling for perceived stress (c′ = 3.79, SE = 0.48, *p* < 0.001, 95% CI [2.84, 4.75]), indicating that resources operate through pathways beyond stress reduction.

Most importantly, the indirect effect through perceived stress was not statistically significant (ab = 0.19, 95% bootstrap CI [−0.17, 0.62]), providing no evidence for mediation in this sample. The confidence interval includes zero, indicating that stress does not carry the beneficial effects of psychological resources to life satisfaction outcomes. Despite the absence of mediation, the overall model explained substantial variance in life satisfaction (R^2^ = 0.35), suggesting that psychological resources are a powerful predictor of employee well-being through alternative mechanisms warranting further investigation.

### 4.5 Country-specific analyses

Separate mediation analyses within each country revealed consistent patterns. In South Africa, the total effect was significant (*c* = 4.62, SE = 0.60, *p* < 0.001), as was the a-path (*a* = −0.88, SE = 0.22, *p* < 0.001), but the b-path remained non-significant (*b* = −0.28, SE = 0.30, *p* = 0.350). The indirect effect was not significant (95% CI [−0.244, 0.836]).

Similarly, in India, the total effect (*c* = 3.12, SE = 0.68, *p* < 0.001) and a-path (*a* = −0.69, SE = 0.28, *p* = 0.016) were significant, but the b-path was not (*b* = −0.16, SE = 0.31, *p* = 0.595). The indirect effect was again non-significant (95% CI [−0.400, 0.781]). These findings indicate that the absence of mediation was consistent across both cultural contexts.

**Figure 1:**
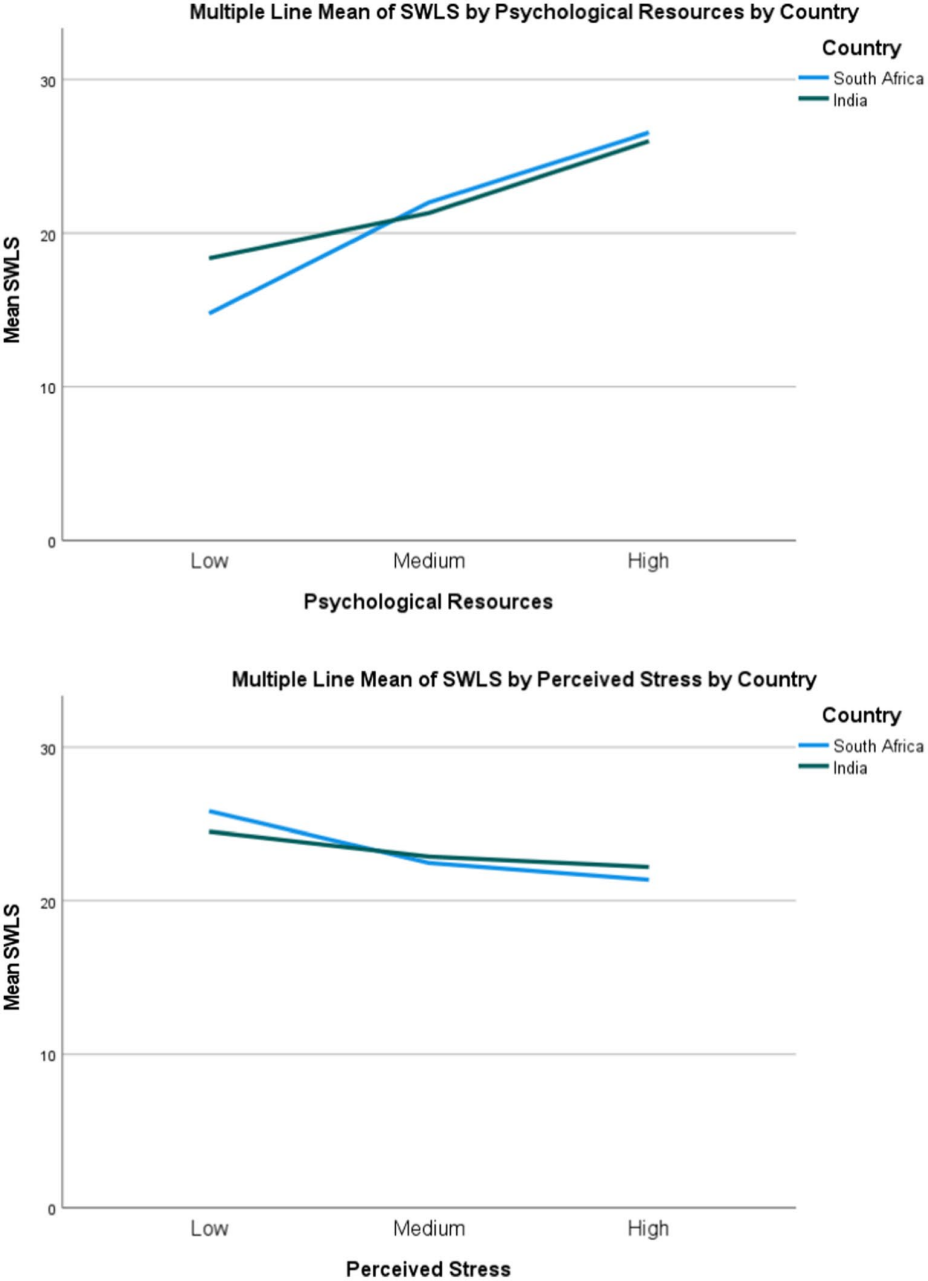
Moderation effects of country on resource–outcome and stress–outcome relationships: **(A)** Resources × Country moderation; **(B)** PSS × Country moderation (authors’ own illustration).

Figure 1 visualizes these moderation effects. Figure 1A depicts the relationship between psychological resources and life satisfaction for each country separately. The near-parallel slopes confirm the non-significant Resources × Country interaction (*p* = 0.165), indicating that the positive association between resources and life satisfaction is equally strong among South African and Indian employees. Similarly, Figure 1B shows that the relationship between perceived stress and life satisfaction (controlling for resources) does not differ by country (PSS × Country: *p* = 0.782), with both countries showing weak, non-significant stress effects. These visual representations reinforce the statistical finding of cross-cultural pathway equivalence.

### 4.6 Cross-cultural moderation analysis

To test whether resilience pathways differed by country, we examined moderation effects using standardized variables. The Resources × Country interaction was not statistically significant (*b* = −0.20, SE = 0.143, *p* = 0.165, 95% CI [−0.48, 0.08]), indicating that the relationship between psychological resources and life satisfaction did not differ significantly between countries. Similarly, the PSS × Country interaction was non-significant (*b* = 0.039, SE = 0.142, *p* = 0.782, 95% CI [−0.24, 0.32]).

The final moderation model explained 35.8% of the variance in life satisfaction (R^2^ = 0.358). The main effect of psychological resources was large (*b* = 0.64, SE = 0.097, *p* < 0.001), while the main effect of perceived stress was small and non-significant (*b* = −0.093, SE = 0.097, *p* = 0.34). Table 7 presents the complete moderation analysis results, demonstrating consistent pathway strength across countries. The non-significant Resources × Country interaction (*p* = 0.165) supports the functional equivalence of resilience mechanisms across these culturally distinct contexts.

**Table 7:** Cross-cultural moderation analysis—resources and stress effects on life satisfaction by country.

Effect	Coefficient	SE	*p*-Value	95% CI
Main effect: Resources	0.64	0.097	< 0.001	[0.45, 0.83]
Main effect: Stress	−0.093	0.097	0.34	[−0.29, 0.09]
Main effect: Country	−0.019	0.134	0.88	[−0.28, 0.25]
Resources * Country	−0.20	0.143	0.165	[−0.48, 0.08]
Stress * Country	0.039	0.142	0.782	[−0.24, 0.32]

R^2^ = 0.36. *N* = 152. Model R^2^ = 0.358 (35.8% variance explained). Analysis used standardized predictors with moderation tested via interaction terms. The non-significant interaction effects (Resources × Country: *p* = 0.165; Stress × Country: *p* = 0.782) indicate that resource and stress pathways do not differ significantly between South African and Indian samples, supporting the cross-cultural generalizability of psychological resource mechanisms. ***p < 0.001.

### 4.7 Supplementary analyses

Including demographic covariates (age, sex) did not alter the pattern of results or improve model fit; therefore, they were excluded for parsimony. Sensitivity analyses using structural equation modeling, with WEMWBS, the Flourishing Scale, and UWES-3 as indicators of a latent psychological resources factor, replicated the main findings: a strong direct pathway to life satisfaction with no significant indirect effect through perceived stress.

### 4.8 Qualitative findings

The qualitative analysis identified culturally distinct yet theoretically convergent themes that explain the quantitative patterns observed. Four themes emerged from the South African data and three from the Indian data, all of which demonstrated clear connections to the statistical findings.

#### 4.8.1 South African themes

##### 4.8.1.1 Ubuntu as collective resilience resource

Participants conceptualized resilience as fundamentally relational, emphasizing the Ubuntu philosophy as an active psychological resource. One participant explained: *“I fund two village children’s schooling because others funded mine. This gives me strength even when I’m struggling.”* This collective orientation transforms individual hardship into meaningful purpose, functioning as what participants described as a “social reserve tank.” Ubuntu represents more than a cultural value; it serves as a practical resource that directly contributes to life satisfaction by enhancing meaning and agency.

##### 4.8.1.2 Dual nature of family support systems

Family relationships simultaneously served as crucial buffers and significant stressors. Participants described complex dynamics in which the family provided essential support while creating substantial demands. One single mother noted: *“The office feels like the only space to breathe because home never stops demanding.”* This duality particularly affected women, who described managing extensive domestic responsibilities alongside professional demands. These accounts illuminate why sex differences emerged in well-being scores, even though perceived stress levels remained similar across sexes.

##### 4.8.1.3 Constrained masculine expression and mental health

Male participants, particularly Black men, described restrictive masculine norms that prohibited help-seeking behavior. As one participant stated, *“In our society, it is very difficult to talk about men’s mental health. Otherwise, we are labelled as weak.”* This cultural constraint helps explain why perceived stress did not mediate the resources–satisfaction relationship; men may systematically under-report stress or manage it through socially invisible coping strategies.

##### 4.8.1.4 Systemic economic and security threats

Participants identified chronic environmental stressors, including economic uncertainty, crime exposure, and infrastructure failures (e.g., load shedding). These conditions created what participants described as “always being on guard,” requiring constant vigilance that depleted cognitive and emotional resources. Black professionals additionally described “Black tax” obligations that reduced the protective effects of higher income, illustrating how structural inequalities moderate resource–outcome relationships.

#### 4.8.2 Indian themes

##### 4.8.2.1 Technology sector pressures and future insecurity

Participants described intense delivery pressures, combined with job insecurity related to artificial intelligence. One junior developer explained: *“AI anxiety shows up in daily choices: fewer breaks, longer hours, staying billable. I get dreams of work only*.*”* This chronic workplace stress was normalized rather than actively addressed, supporting the finding that stress correlated with resources but did not mediate life satisfaction outcomes.

##### 4.8.2.2 Aspirational mobility and infrastructure friction

Participants expressed pride in India’s technological advancement and optimism about career prospects, which served as psychological resources that directly contributed to life satisfaction. However, poor urban infrastructure, lengthy commutes, and housing costs created daily friction that tempered this optimism. This tension illustrates how both positive and negative factors can coexist within an individual’s experience.

##### 4.8.2.3 Geographic separation and social resource depletion

Migration to Mumbai for career advancement necessitated separation from family and established social networks. Participants described this as a “quiet drain” on emotional resources, with video communication providing insufficient replacement for proximal social support. This geographic displacement helps explain why stress remains present but remains disconnected from changes in life satisfaction.

#### 4.8.3 Mixed-methods integration and theoretical implications

Table 2 presents a systematic triangulation of quantitative patterns with qualitative explanations. The integration reveals three key theoretical insights:

First, psychological resources appear to influence life satisfaction through direct pathways involving meaning, purpose, and identity rather than through stress-reduction mechanisms. Both Ubuntu practices in South Africa and the technology sector in India provide immediate sources of meaning in life that enhance satisfaction regardless of stress levels.

Second, perceived stress operates as an ambient background rather than an active mediator in these contexts. Stress was normalized, constrained by disclosure norms, or managed through culturally specific coping strategies that maintained its presence while preventing its translation into reduced life satisfaction.

Third, structural and cultural factors systematically moderate individual psychological processes. Sex role expectations, economic obligations, and cultural norms around emotional expression shape how resources translate into outcomes, suggesting that resilience interventions must account for contextual constraints on individual agency.

These findings extend the COR theory by demonstrating that resource–outcome pathways may operate through multiple mechanisms simultaneously, with cultural meaning-making potentially superseding stress reduction as the primary pathway to well-being in certain contexts.

## 5 Discussion

### 5.1 Summary of key findings and hypothesis evaluation

This cross-national study examined whether psychological resources directly or indirectly influence life satisfaction by reducing perceived stress among employees in South Africa and India. Three key findings emerged from our mixed-methods analysis, which we now evaluate against our hypotheses and position within existing literature.

Hypothesis 1 predicted that psychological resources would be positively associated with life satisfaction in both countries. This hypothesis was strongly supported. Psychological resources demonstrated a robust positive association with life satisfaction across both cultural contexts. In the simple pooled regression, the standardized coefficient was *β* = 0.71 (*b* = 3.79, SE = 0.48, *p* < 0.001); in the full moderation model including country and interaction terms, the main effect was *b* = 0.64 (SE = 0.097, *p* < 0.001). Both models converge to confirm a large, significant direct pathway from resources to life satisfaction. This finding aligns with prior research in both regions: Rothmann (2008) found that work engagement is a dimension of well-being among South African employees, and Zhou et al. (2023) demonstrated similar resource–outcome relationships (resilience positively predicts engagement) among Chinese healthcare workers. Our contribution is the first direct cross-national comparison confirming functional equivalence across South African and Indian contexts. These findings also carry implications for employee resilience theory. As noted by Hartmann et al. (2020) and Raetze et al. (2021), psychological resources are key antecedents to resilient functioning at work, and our evidence that resource portfolios directly predict life satisfaction in non-Western contexts extends this argument beyond the predominantly Western samples on which these reviews were based.

Hypothesis 2 predicted partial mediation via perceived stress. This hypothesis was not supported. Perceived stress did not significantly mediate the resources–satisfaction relationship in either country, despite significant negative associations between resources and stress. This diverges from traditional stress-and-coping models (Lazarus and Folkman, 1984) but aligns with Arnold et al. (2007), who showed that transformational leadership enhanced employee well-being primarily through meaningful work rather than through stress reduction.

Hypothesis 3 predicted cross-national differences in pathway strength. This hypothesis was not supported; pathway coefficients did not differ significantly across countries (Resources × Country, *p* = 0.165). This finding contradicts expectations based on individualism–collectivism frameworks, which typically suggest that cultural context produces significant variability in psychological and behavioral outcomes (Medvedev et al., 2024). For example, recent cross-national evidence found that the “money advantage” (the motivating effect of monetary over psychological incentives) was significantly stronger in individualistic Western cultures than in more collectivist contexts such as India and South Africa (Medvedev et al., 2024).

Our qualitative findings revealed why pathways remain equivalent despite cultural differences: psychological resources operate through meaning-making mechanisms (Ubuntu-based purpose in South Africa and aspirational mobility pride in India), demonstrating that cultural content differs while psychological mechanisms remain constant.

### 5.2 Theoretical implications and mechanism clarification

#### 5.2.1 Direct resource pathways and COR theory extension

**Figure 2:**
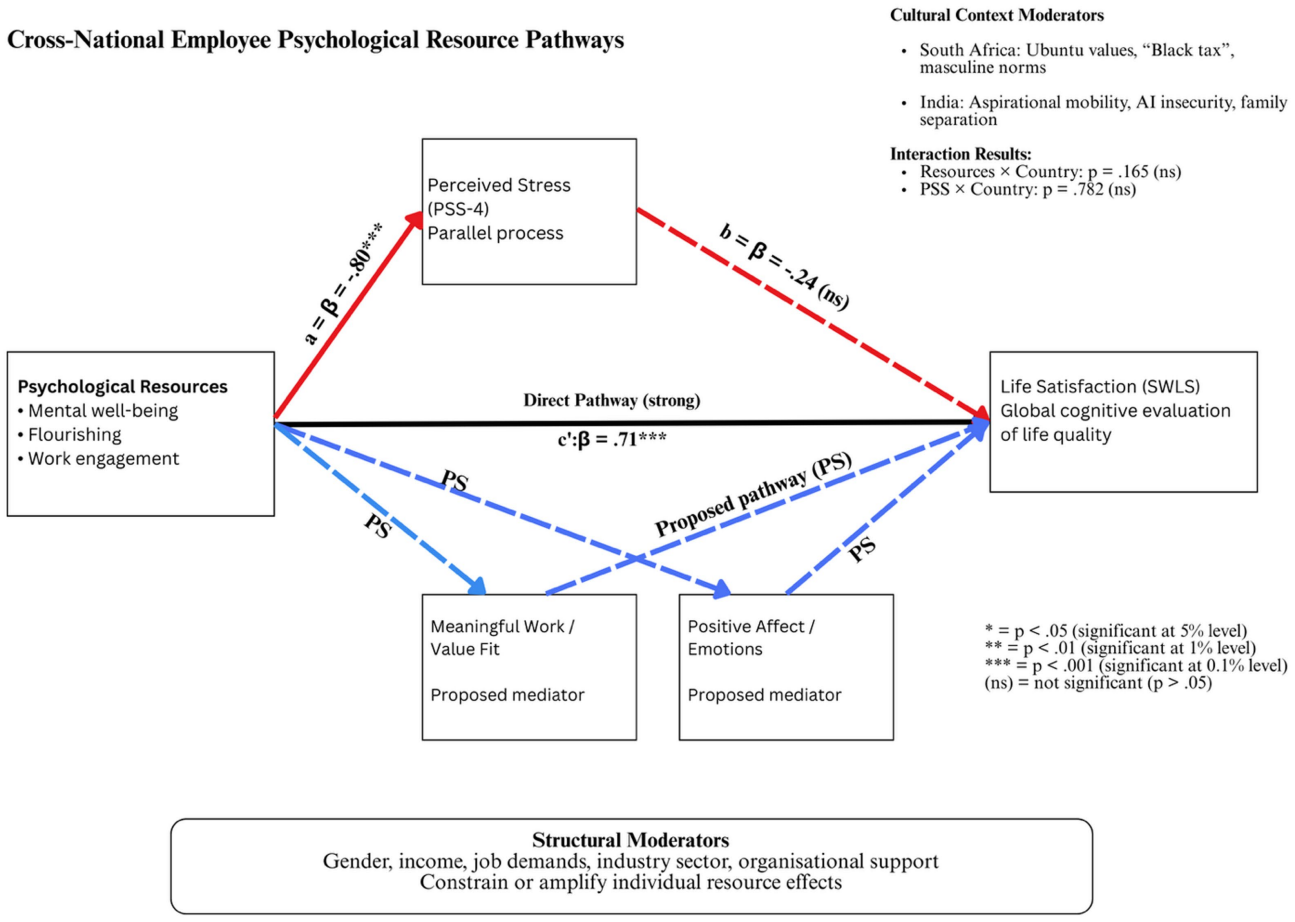
Psychological resource pathways to life satisfaction: cross-national mediation and moderation model (authors’ own illustration).

Our findings extend the COR theory by demonstrating that resource–outcome relationships may operate through multiple simultaneous mechanisms rather than sequential mediation chains. The strong direct pathway from psychological resources to life satisfaction (β = 0.71, *p* < 0.001; Figure 2) remained robust even when controlling for perceived stress, suggesting that resources enhance well-being through pathways beyond stress buffering. This aligns with evidence indicating that meaningful work and positive affect are critical drivers of well-being, influencing personal resource pools and cognitive life satisfaction outcomes (Arnold et al., 2007; Csordás et al., 2022).

Qualitative findings illuminate why direct pathways dominate in our sample. Ubuntu practices in South Africa and the technology sector in India both serve as meaning-making resources that directly enhance life evaluations by fostering greater purpose and identity affirmation. This supports emerging theoretical perspectives suggesting that eudaimonic well-being pathways—focused on purpose, personal growth, and social contribution—may be more consistent with long-term orientation and potentially supersede hedonic pathways in certain cultural contexts (Joshanloo et al., 2021).

#### 5.2.2 Stress as a parallel process rather than a mediator

The non-significant mediation by perceived stress challenges traditional stress-and-coping models that position stress reduction as the primary mechanism through which resources enhance well-being. Instead, our data suggest that stress functions as a parallel process, correlating with resources but not carrying their effect to life satisfaction. Three theoretical explanations support this pattern.

First, in relatively healthy employee populations with moderate stress levels, stress may function as an ambient background rather than an active determinant of life satisfaction. This aligns with set-point theory, suggesting that life satisfaction is influenced more by stable personality factors and meaning systems than by fluctuating stressors (Diener et al., 1985; Seligman, 2011). Second, cultural factors may constrain stress disclosure and expression, particularly among male participants who described masculine norms prohibiting help-seeking behavior. This measurement constraint may artificially weaken mediation pathways. Third, the limited reliability of PSS-4 (*α* = 0.62) likely attenuated the b-path in our mediation model, although sensitivity analyses suggest this alone cannot account for the negligible indirect effect.

### 5.3 Cross-cultural generalizability and functional equivalence

#### 5.3.1 Cultural content versus psychological mechanisms

A striking finding was the absence of significant country-moderation effects, suggesting functional equivalence in resilience mechanisms across distinct cultural contexts. This challenges the assumption that individualistic and collectivistic cultures necessarily follow different well-being pathways. Our mixed-methods integration reveals that while the content of meaning-making differs culturally (Ubuntu *vs*. technological advancement), the psychological mechanisms remain consistent: resources enhance life satisfaction through direct meaning and purpose pathways rather than stress reduction.

This functional equivalence has important theoretical implications for cross-cultural psychology. It suggests that certain fundamental psychological processes, particularly those linking personal resources to life evaluations, may be more culturally universal than previously assumed. However, this universality operates at the mechanism level rather than the content level, requiring culturally adaptive interventions that target universal processes through culturally specific content.

#### 5.3.2 Structural moderators and contextual constraints

Although the country did not moderate pathway strength, qualitative findings reveal important structural moderators that warrant future investigation. Sex differences in well-being emerged clearly in the South African data, explained by gendered domestic labor expectations and “Black tax” obligations that limit the benefits of resources for women and Black professionals, respectively. These patterns suggest that individual psychological resources may be necessary but insufficient for well-being when structural inequalities constrain their translation into positive outcomes.

### 5.4 Integrative theoretical framework

Building on our empirical findings and theoretical analysis, we propose an integrative cross-national psychological resource framework (Figure 2) that synthesizes quantitative pathways with qualitative mechanisms and cultural moderators.

This integrative framework synthesizes our empirical findings with the COR theory and cross-cultural psychology principles. The framework demonstrates that psychological resources influence life satisfaction primarily through direct pathways rather than stress-mediated routes. The model incorporates three levels of analysis: individual psychological processes (resources → outcomes), cultural contextual factors that shape resource content without altering mechanism strength, and structural moderators that constrain or amplify individual resource effects.

### 5.5 Practical implications for organizations

#### 5.5.1 Resource-focused intervention design

Our findings suggest that organizational well-being interventions should prioritize resource building over stress management as the primary pathway to enhanced life satisfaction. This aligns with emerging evidence that workplace well-being programs demonstrate effectiveness when they focus on enhancing positive psychological states rather than merely reducing negative ones (Cameron et al., 2025; Hendriks et al., 2020; Shekhar et al., 2025b; Shekhar et al., 2025a). Specifically, organizations should invest in programs that enhance mental well-being, foster meaningful work experiences, and increase work engagement through job crafting and strengths-based approaches.

The cross-cultural consistency in pathway strength suggests that multinational organizations can implement similar intervention mechanisms across diverse cultural contexts while adapting specific content to local meaning systems. In South African contexts, interventions might incorporate community service components that leverage Ubuntu values, while in Indian contexts, they might emphasize career development and technological skill building that align with aspirational mobility narratives.

##### 5.5.1.1 Addressing structural constraints

Importantly, our qualitative findings reveal that individual resource-building interventions may be insufficient when structural inequalities constrain their translation into positive outcomes. Organizations must address systemic barriers—such as gendered work expectations, income inequalities, and cultural norms that limit help-seeking—alongside individual interventions. This multi-level approach aligns with recent calls for systemic approaches to workplace well-being that address both individual capacities and organizational structures (Global Wellness Institute, 2025).

### 5.6 Limitations and future research directions

This pilot study has several important limitations that constrain generalizability and causal inference. The modest sample size (*N* = 152) limited statistical power for detecting small mediation effects and precluded sophisticated multi-group structural equation modeling. The cross-sectional design prevents causal inferences about the directionality of relationships between resources, stress, and life satisfaction. Additionally, the documented reliability limitations of PSS-4 may have artificially weakened mediation pathways, although sensitivity analyses suggest this cannot fully account for the null indirect effects observed. In addition, the single-source, self-report design introduces potential CMB, although procedural safeguards (independent administration, confidentiality assurances, varied response formats) and Harman’s test results suggest this concern is mitigated to some extent.

A further limitation concerns the measurement model. We did not conduct a full confirmatory factor analysis with individual item loadings due to sample size constraints, as the participant-to-parameter ratio would be inadequate for stable CFA estimation (typically requiring *N* > 300 for complex models; Kline, 2016). All scales employed have been extensively validated in prior research, including in South African and Indian contexts (see Section 3.4), but measurement invariance across countries was not formally tested. Future studies should employ larger samples to enable full CFA-based measurement validation and cross-national invariance testing.

Future research should address these limitations through several methodological advances. Longitudinal designs with multiple time points would enable testing of dynamic resource accumulation and depletion processes predicted by the COR theory. Larger multi-site samples would permit adequately powered tests of cultural moderation effects and structural equation modeling with latent variables. Investigation of multiple mediators simultaneously—including meaningful work, positive affect, and self-efficacy—would clarify which mechanisms primarily account for resource–outcome relationships across cultural contexts.

Additionally, future studies should incorporate objective measures of workplace stressors and performance outcomes to complement self-report measures of well-being. Intervention studies testing resource-building programs would provide stronger evidence for causal pathways and practical effectiveness across cultural contexts. Finally, investigation of additional moderators—including job demands, leadership quality, and organizational support—would enhance understanding of when and how individual resources translate most effectively into positive outcomes.

### 5.7 Conclusion

This cross-national study directly addressed three critical gaps identified in the literature. First, we provided the first empirical comparison of resource–stress–life satisfaction pathways between South African and Indian employees, demonstrating cross-cultural pathway equivalence. Second, by operationalizing psychological resources as a composite portfolio rather than separate constructs, we demonstrated that holistic resource accumulation predicts life satisfaction more strongly than individual resources alone. Third, we explicitly tested rather than assumed stress mediation, revealing that resources operate primarily through direct pathways (meaning and purpose) rather than stress buffering—a finding with important implications for both COR theory and intervention design.

This cross-national study provides evidence regarding how psychological resources translate into employee life satisfaction among South African and Indian employees. Three key empirical findings emerged. First, psychological resources demonstrated a robust, direct association with life satisfaction in both countries (*b* = 3.79, *p* < 0.001, *R*^2^ = 0.358), supporting Hypothesis 1 and extending COR theory beyond Western samples. Second, contrary to Hypothesis 2, perceived stress did not mediate this relationship despite significant negative associations between resources and stress (*a* = −0.80, *p* < 0.001). The non-significant *b*-path (*p* = 0.259) and the bootstrap confidence interval that includes zero indicate that stress operates as a parallel process rather than an explanatory mechanism. Third, pathway strengths were statistically equivalent across countries (Hypothesis 3 not supported), demonstrating functional equivalence of resource–outcome mechanisms despite distinct cultural contexts.

These findings necessitate theoretical refinement of COR models applied to employee well-being. Rather than operating primarily through stress buffering, psychological resources appear to enhance life satisfaction through direct pathways involving meaning, purpose, and identity affirmation—mechanisms illuminated by our qualitative findings but requiring explicit measurement in future quantitative research. The cross-cultural consistency observed at the mechanism level (equivalent pathway coefficients) despite cultural variation in resource content (Ubuntu *vs*. aspirational pride) suggests that COR theory successfully predicts *how* resources operate. However, it requires cultural contextualization to specify *which* resources matter most in particular settings.

Practically, organizations in South Africa, India, and comparable emerging economies should prioritize resource-building interventions (enhancing mental well-being, fostering meaningful work, increasing engagement) over stress management programs as primary pathways to employee life satisfaction. Although stress reduction remains important for health outcomes, our findings suggest that it is insufficient as the sole mechanism for enhancing well-being. The cross-national consistency in mechanism strength further implies that multinational organizations can implement similar intervention structures across diverse cultural contexts while adapting specific content to align with local meaning systems—a pragmatic strategy for global well-being initiatives.

Future research should test alternative mediators (meaningful work, positive affect, self-efficacy) through adequately powered longitudinal designs, enabling stronger causal inference and dynamic testing of resource accumulation processes predicted by COR theory.

## Data Availability

The raw data supporting the conclusions of this article will be made available by the authors, without undue reservation.
