# Supplementary material for: Psychological resource pathways to life satisfaction in South Africa and India: a cross-national pilot study with implications for employee resilience
Source: Front Psychol. 2026 Apr 14;17:1706890. doi: 10.3389/fpsyg.2026.1706890 (PMC13121078; doi:10.3389/fpsyg.2026.1706890)
Supplement: Supplementary file 1 [file Data_Sheet_1.docx]

**Supplementary Table S1:** **Consolidated Criteria for Reporting Qualitative Research (COREQ) Checklist**

| **#** | **Item** | **Guide question** | **Study answer (editable)** | **Location in manuscript** |
| --- | --- | --- | --- | --- |
| **Domain 1: Research team & reflexivity** | | | | |
| 1 | Interviewer/facilitator | Which author conducted the interviews/FGD? | Lead researcher (AS) conducted the FGD and all interviews. | Methods → Qualitative interviews |
| 2 | Credentials | What were the researcher’s credentials? | PhD candidate (University of Johannesburg) at the time of data collection. | Methods → Research design |
| 3 | Occupation | What was their occupation at the time? | Part-time doctoral researcher. | Methods → Participants & sampling |
| 4 | Gender | Gender of interviewer | Male. | Methods → Researcher positionality note |
| 5 | Experience & training | Interviewer’s qualitative experience | Trained in mixed methods as part of PhD programme | Methods → Research design |
| 6 | Relationship established | Was a relationship established prior to study? | Yes. Researcher was an employee at one of the two companies and had interacted with participants via the well-being programme. | Methods → Participants & sampling |
| 7 | Participant knowledge of the interviewer | What did participants know? | Study on well-being, confidentiality, and consent | Data collection → Survey/FGD/Interview procedures |
| 8 | Interviewer characteristics | Report characteristics, assumptions, reflexivity | Reflexive note acknowledged the interviewer’s position. | Methods → Researcher positionality note |
| **Domain 2: Study design** | | | | |
| 9 | Methodological orientation | What theoretical/analytic approach? | Reflexive thematic analysis (Braun & Clarke, 2019). | Methods → Data analysis (qual) |
| 10 | Sampling | How were participants selected? | Purposive maximum-variation sampling across gender, race, generation, role, and qualification. | Methods → Participants & sampling |
| 11 | Method of approach | How were participants contacted? | HR admin scheduled times in participants’ calendars. | Methods → Data collection (qual) |
| 12 | Sample size | How many participants? | Different number of participants for two different methods | Methods |
| 13 | Non-participation | Refusals/dropouts? | No refusals recorded | Methods → Participants & sampling |
| 14 | Setting of data collection | Where were data collected? | Online and face to face based on availability | Methods → Data collection (qual) |
| 15 | Presence of non-participants | Anyone else present? | No. Only participant and researcher were present. | Methods → Data collection (qual) |
| 16 | Description of sample | Key sample characteristics | Diverse by race, gender, age, qualifications. | Methods |
| 17 | Interview guide | Were guides provided/piloted? | Semi-structured guide developed from literature and survey patterns; piloted informally; prompts adapted iteratively. | Methods → Data collection (qual) |
| 18 | Repeat interviews | Were repeats carried out? | No repeat interview. Multiple informal sessions were held towards intervention. | Methods → Qualitative interviews |
| 19 | Audio/visual recording | Were sessions recorded? | No. Participants were more comfortable and candid when call was not recorded. | Methods → Data collection (qual) |
| 20 | Field notes | Were field notes made? | Yes. Analytic field notes captured context, non-verbal cues, and reflexive impressions. | Methods → Data collection (qual) |
| 21 | Duration | Interview/FGD length | FGD ≈60+ minutes; interviews ≈30+ minutes each. | Methods → Qualitative interviews |
| 22 | Data saturation | Was saturation discussed? | Yes. Recruitment ceased when thematic saturation was reached (recurring patterns; no new substantive insights). | Methods → Participants & sampling |
| 23 | Transcripts returned | Were transcripts returned for comment? | No transcript returns. Informal clarification/participant checking occurred within sessions. | Methods → Trustworthiness |
| **Domain 3: Analysis & findings** | | | | |
| 24 | Number of data coders | How many analysts/coders? | Primary coder: lead researcher; credibility supported via peer debriefs with HR/academic peers. | Methods → Data analysis (qual) |
| 25 | Description of coding tree | Was a coding tree described? | Inductive codebook developed iteratively; higher-order themes generated from clustered codes | Methods → Data analysis (qual) |
| 26 | Derivation of themes | How were themes developed? | Themes were developed iteratively from inductive codes, reviewed against the full corpus, and named to capture patterned meaning at a latent level. | Methods → Data analysis (qual) |
| 27 | Software | What software was used? | Manual analysis (no CAQDAS); spreadsheets/word-processing aided organization. | Methods → Data analysis (qual) |
| 28 | Participant checking | Did participants provide feedback on findings? | Yes; resonance was checked informally through multiple informal interactions. | Methods → Trustworthiness |
| 29 | Quotations presented | Are participant quotes presented? | Yes. | Results → Themes |
| 30 | Data–findings consistency | Is there consistency between data and findings? | Yes. Integration with survey patterns demonstrate alignment. | Results → Integration section |
| 31 | Clarity of major themes | Are major themes clear? | Yes. Four themes are explicitly titled, introduced, and substantiated. | Results → Themes 1–4 |
| 32 | Clarity of minor themes | Are diverse cases/nuances described? | Yes. | Results → Themes; Discussion |

**Supplementary Table S2: Individual Resource Mediation Analyses**

**Model 2a: WEMWBS → PSS → SWLS:**

The following table shows Mediation Analysis Results - Psychological Resource (WEMEBS), Perceived Stress, and Life Satisfaction.

| **Table 1: Mediation Analysis Results - WEMWBS, Perceived Stress, and Life Satisfaction** | | | | |
| --- | --- | --- | --- | --- |
| **Path/Effect** | **Coefficient** | **SE** | **p-value** | **95% CI** |
| Total Effect (c) | 0.39 | 0.05 | <0.001 | [0.28, 0.49] |
| a-path | -0.09 | 0.019 | <0.001 | [-0.13, -0.06] |
| b-path | -0.29 | 0.23 | 0.19 | [-0.74, 0.15] |
| Direct Effect (c′) | 0.36 | 0.06 | <0.001 | [0.25, 0.46] |
| Indirect Effect (ab) | 0.027 |  |  | [-0.018, 0.07] |

$$\boldsymbol{R}^{\boldsymbol{2}}\boldsymbol{=}\boldsymbol{0}\boldsymbol{.}\boldsymbol{27}$$

**Model 2b: Flourish → PSS → SWLS:**

The following table shows Mediation Analysis Results - Psychological Resource (Flourish), Perceived Stress, and Life Satisfaction.

| **Table 2: Mediation Analysis Results - Flourish, Perceived Stress, and Life Satisfaction** | | | | |
| --- | --- | --- | --- | --- |
| **Path/Effect** | **Coefficient** | **SE** | **p-value** | **95% CI** |
| Total Effect (c) | 0.59 | 0.07 | <0.001 | [0.46, 0.73] |
| a-path | -0.09 | 0.026 | <0.001 | [-0.15, -0.04] |
| b-path | -0.36 | 0.21 | 0.08 | [-0.77, 0.04] |
| Direct Effect (c′) | 0.56 | 0.07 | <0.001 | [0.42, 0.69] |
| Indirect Effect (ab) | 0.035 |  |  | [-0.006, 0.09] |

$$\boldsymbol{R}^{\boldsymbol{2}}\boldsymbol{=}\boldsymbol{0}\boldsymbol{.}\boldsymbol{35}$$

**Model 2c: UWES → PSS → SWLS:**

The following table shows Mediation Analysis Results - Psychological Resource (UWES), Perceived Stress, and Life Satisfaction.

| **Table 3: Mediation Analysis Results - UWES, Perceived Stress, and Life Satisfaction** | | | | |
| --- | --- | --- | --- | --- |
| **Path/Effect** | **Coefficient** | **SE** | **p-value** | **95% CI** |
| Total Effect (c) | 2.46 | 0.42 | <0.001 | [1.63, 3.29] |
| a-path | -0.49 | 0.15 | 0.0012 | [-0.79, -0.19] |
| b-path | -0.54 | 0.23 | 0.019 | [-0.98, -0.09] |
| Direct Effect (c′) | 2.19 | 0.43 | <0.001 | [1.34, 3.05] |
| Indirect Effect (ab) | 0.27 |  |  | [0.02, 0.63] |

$\boldsymbol{R}^{\boldsymbol{2}}\boldsymbol{=}\boldsymbol{0}\boldsymbol{.}$**21**

**Supplementary Table S3: Composite Reliability (CR) and Average Variance Extracted (AVE) by Scale**

| **Scale** | **Composite Reliability (CR)** | **Average Variance Extracted (AVE)** |
| --- | --- | --- |
| WEMWBS | .900 | .403 |
| Flourishing | .856 | .436 |
| UWES-3 | .825 | .614 |
| SWLS | .858 | .548 |
| PSS-4 | .501 | .232 |
|  | | |
| Note. Recommended thresholds: CR > .70; AVE > .50. PSS-4 fell below both thresholds, reflecting measurement limitations of the brief 4-item version (acknowledged in Section 5.6). WEMWBS and Flourishing met the CR threshold; AVE was marginally below .50. | | |

**Supplementary Table S4: Standardised CFA Factor Loadings for All Scales**

| **Item** | **EST** | **SE** | **z** | **p-value** | **CI Lower** | **CI Upper** | **Std.lv** | **Std. Loading** |  |
| --- | --- | --- | --- | --- | --- | --- | --- | --- | --- |
|  | | | | | | | | | |
| WEMWBS1 | 1 | 0 | NA | NA | 1 | 1 | 0.502 | 0.580 |  |
| WEMWBS2 | 0.899 | 0.151 | 5.947 | <.001 | 0.603 | 1.196 | 0.452 | 0.583 |  |
| WEMWBS3 | 0.881 | 0.160 | 5.507 | <.001 | 0.568 | 1.195 | 0.443 | 0.527 |  |
| WEMWBS4 | 0.773 | 0.162 | 4.783 | <.001 | 0.456 | 1.089 | 0.388 | 0.443 |  |
| WEMWBS5 | 1.268 | 0.200 | 6.328 | <.001 | 0.875 | 1.660 | 0.637 | 0.634 |  |
| WEMWBS6 | 0.681 | 0.138 | 4.941 | <.001 | 0.411 | 0.951 | 0.342 | 0.460 |  |
| WEMWBS7 | 0.989 | 0.149 | 6.651 | <.001 | 0.698 | 1.281 | 0.497 | 0.681 |  |
| WEMWBS8 | 1.524 | 0.205 | 7.443 | <.001 | 1.123 | 1.926 | 0.766 | 0.813 |  |
| WEMWBS9 | 1.049 | 0.176 | 5.960 | <.001 | 0.704 | 1.394 | 0.527 | 0.584 |  |
| WEMWBS10 | 1.313 | 0.180 | 7.298 | <.001 | 0.960 | 1.665 | 0.659 | 0.787 |  |
| WEMWBS11 | 0.953 | 0.158 | 6.028 | <.001 | 0.643 | 1.262 | 0.479 | 0.593 |  |
| WEMWBS12 | 1.024 | 0.185 | 5.523 | <.001 | 0.661 | 1.388 | 0.514 | 0.529 |  |
| WEMWBS13 | 1.320 | 0.196 | 6.731 | <.001 | 0.935 | 1.704 | 0.663 | 0.694 |  |
| WEMWBS14 | 1.281 | 0.173 | 7.402 | <.001 | 0.942 | 1.620 | 0.644 | 0.805 |  |
|  | | | | | | | | | |
| SWLS1 | 1 | 0 | NA | NA | 1 | 1 | 1.130 | 0.750 |  |
| SWLS2 | 0.906 | 0.091 | 10.002 | .000 | 0.729 | 1.084 | 1.024 | 0.807 |  |
| SWLS3 | 1.115 | 0.101 | 11.085 | .000 | 0.918 | 1.312 | 1.260 | 0.911 |  |
| SWLS4 | 0.878 | 0.104 | 8.479 | .000 | 0.675 | 1.082 | 0.993 | 0.693 |  |
| SWLS5 | 0.964 | 0.132 | 7.313 | <.001 | 0.706 | 1.222 | 1.089 | 0.604 |  |
|  | | | | | | | | | |
| Flourish1 | 1 | 0 | NA | NA | 1 | 1 | 0.766 | 0.693 |  |
| Flourish2 | 0.847 | 0.138 | 6.122 | .000 | 0.576 | 1.119 | 0.649 | 0.550 |  |
| Flourish3 | 1.150 | 0.142 | 8.114 | .000 | 0.872 | 1.428 | 0.881 | 0.750 |  |
| Flourish4 | 0.799 | 0.109 | 7.300 | .000 | 0.585 | 1.014 | 0.612 | 0.665 |  |
| Flourish5 | 0.750 | 0.102 | 7.317 | .000 | 0.549 | 0.951 | 0.574 | 0.666 |  |
| Flourish6 | 0.702 | 0.100 | 7.024 | .000 | 0.506 | 0.898 | 0.538 | 0.637 |  |
| Flourish7 | 1.130 | 0.148 | 7.638 | .000 | 0.840 | 1.420 | 0.866 | 0.699 |  |
| Flourish8 | 0.686 | 0.110 | 6.224 | .000 | 0.470 | 0.902 | 0.526 | 0.559 |  |
|  | | | | | | | | | |
| UWES1 | 1 | 0 | NA | NA | 1 | 1 | 0.931 | 0.714 |  |
| UWES2 | 1.143 | 0.138 | 8.275 | .000 | 0.872 | 1.414 | 1.064 | 0.926 |  |
| UWES3 | 0.891 | 0.108 | 8.217 | .000 | 0.679 | 1.104 | 0.829 | 0.712 |  |
|  | | | | | | | | | |
| PSS1 | 1 | 0 | NA | NA | 1 | 1 | 0.430 | 0.454 |  |
| PSS2 | 0.657 | 0.249 | 2.644 | .008 | 0.170 | 1.144 | 0.282 | 0.417 |  |
| PSS3 | 0.305 | 0.173 | 1.760 | .078 | -0.035 | 0.644 | 0.131 | 0.220 |  |
| PSS4 | 1.273 | 0.527 | 2.417 | .016 | 0.241 | 2.305 | 0.547 | 0.612 |  |
